# Primary Cutaneous Neoplasm With Rhabdomyosarcomatous Differentiation and a Melanoma‐Like Mutational Landscape

**DOI:** 10.1111/cup.14806

**Published:** 2025-03-16

**Authors:** Maximillian A. Weigelt, Shinoj Pattali, Josephine K. Dermawan, Jennifer S. Ko, Karen J. Fritchie, Steven D. Billings

**Affiliations:** ^1^ Department of Pathology and Laboratory Medicine Cleveland Clinic Cleveland Ohio USA; ^2^ Department of Hematology and Oncology Cleveland Clinic Foundation Cleveland Ohio USA

**Keywords:** melanoma, molecular, PD‐1, rhabdomyosarcoma, trans‐differentiated, whole exome sequencing

## Abstract

Malignant melanoma (MM) is notorious for its wide range of morphologic variability. Rarely, MM may lose all melanocytic markers and adopt the morphologic and immunophenotypic characteristics of a different neoplasm in a process known as trans‐differentiation (TMM). Distinguishing TMM from primary cutaneous neoplasms may be challenging and is often dependent on the identification of an adjacent conventional melanoma. In particularly difficult cases, molecular analysis may be helpful; TMMs are known to exhibit highly similar mutational landscapes to conventional melanomas (e.g., mutations in *NF1*, *NRAS*; variable *BRAF* V600E). Herein, we present an exceedingly rare case of likely TMM with rhabdomyosarcomatous differentiation in which high tumor mutational burden (TMB) was an important clue to the diagnosis. An 83‐year‐old woman presented with an 8.2 cm fungating mass on the upper arm. Biopsy revealed a sheet‐like proliferation of mitotically active pleomorphic cells which were positive for myogenin/MyoD1 and negative for S100/SOX10. A diagnosis of epithelioid rhabdomyosarcoma was rendered. Subsequent axillary lymph node metastasis prompted whole exome sequencing, which revealed a molecular signature more indicative of MM, including: high TMB (19 mutations/Mb); ultraviolet mutational signature (i.e., preponderance of C>T base changes); *TERT* promoter mutation; and *ARID2* mutation. After discussion at the interdisciplinary tumor board, a diagnosis of TMM was considered most likely, and the patient was initiated on pembrolizumab. Morphologic features more typical of MM than cutaneous sarcomas, such as tumor‐infiltrating lymphocytes, junctional epidermal tumor nests, and satellitosis, may provide further clues to the accurate diagnosis of TMM, which has important prognostic and therapeutic implications for the patient.

## 1 Introduction

Melanoma is infamous for its wide range of histomorphologic heterogeneity, which has earned it a place among medicine's many “great mimickers.” In rare cases, melanoma may lose all indicators of melanocytic differentiation and adopt the morphologic and immunophenotypic characteristics of an entirely different tissue in a process known as trans‐differentiation [1, 2]. Distinguishing trans‐differentiated melanoma from other primary cutaneous neoplasms can be challenging and is typically dependent on the identification of an adjacent conventional melanoma [2]. However, in rare cases where a conventional component cannot be identified, the molecular landscape may provide the only clue to its true nature [1]. Herein, we present an exceedingly rare case of a primary cutaneous neoplasm with rhabdomyosarcomatous differentiation and a melanoma‐like mutational landscape, which may represent trans‐differentiated melanoma.

## 2 Case Report

**FIGURE 1:**
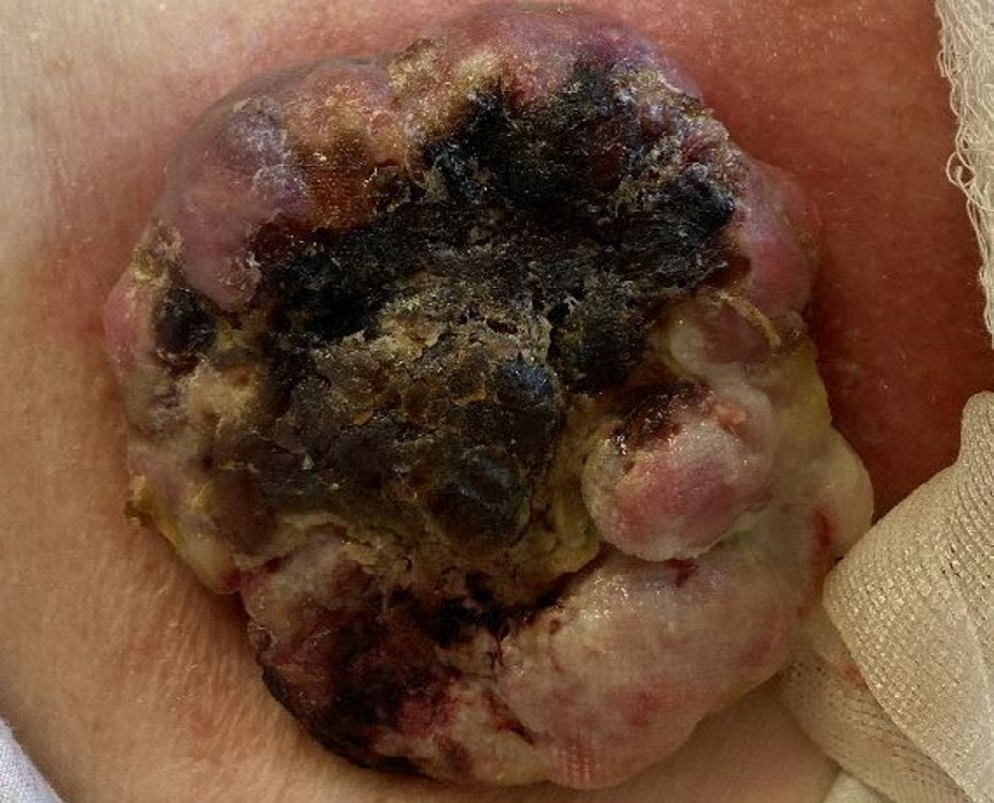
Clinical presentation of a primary cutaneous neoplasm with rhabdomyosarcomatous differentiation. An 83‐year‐old woman presented with an 8.2‐cm fungating mass on the posterolateral deltoid.

An 83‐year‐old woman presented to the surgical clinic with a one‐year history of a slowly growing, intermittently bleeding right arm mass. Physical examination revealed an 8.2 cm fungating mass on the posterolateral aspect of the right deltoid (Figure 1). An incisional biopsy, and later wide excision with axillary lymph node dissection, was performed. Informed consent for the publication of case details was obtained from the patient.

**FIGURE 2:**
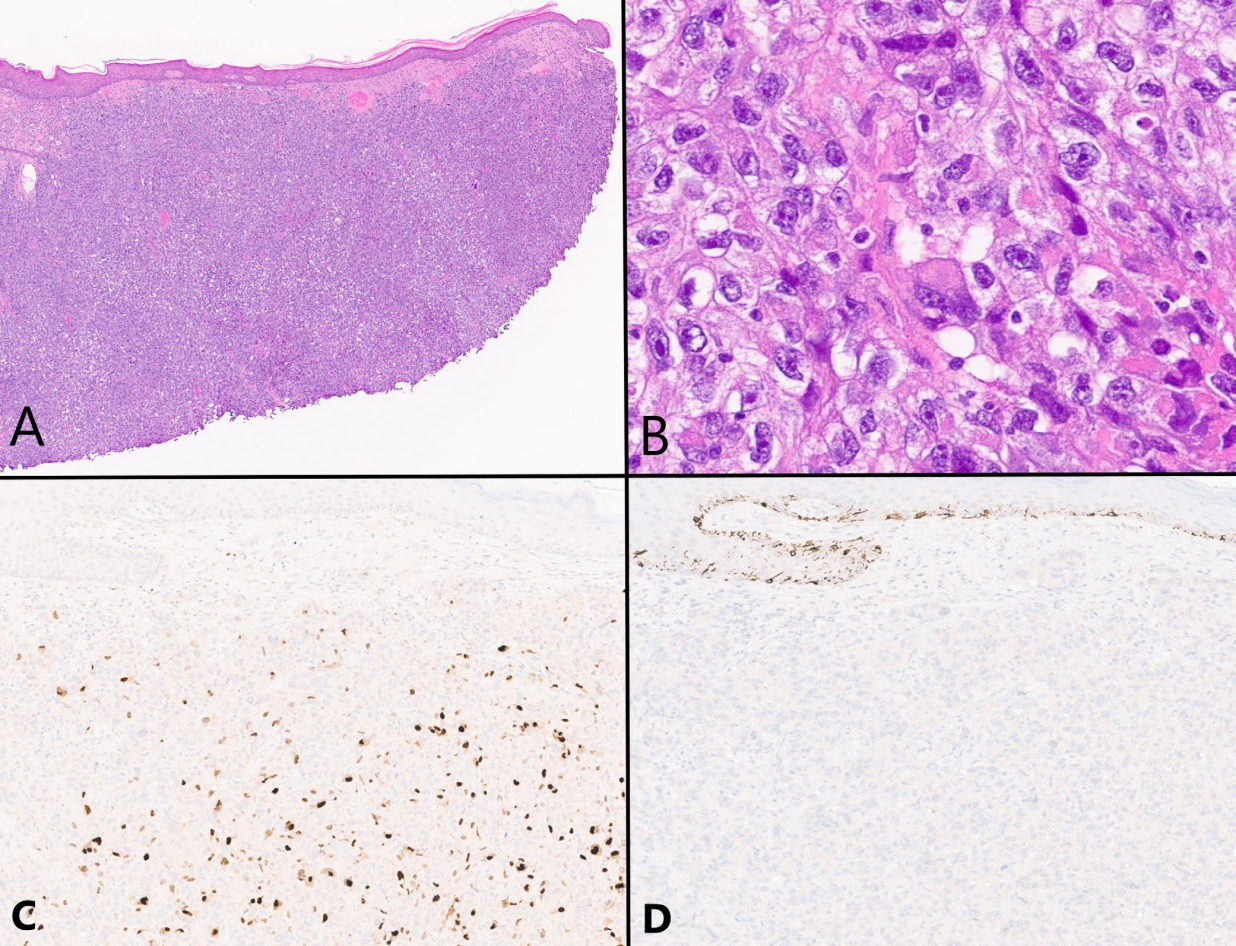
Histologic features of a primary cutaneous neoplasm with rhabdomyosarcomatous differentiation. (A) Low‐power examination revealed a sheet‐like proliferation of cells in the dermis (*H&E, 4*×). (B) The tumor was composed of atypical epithelioid‐to‐rhabdoid cells with enlarged vesicular nuclei, variably prominent nucleoli, and amphophilic cytoplasm (*H&E, 40*×). (C) A myogenin stain was positive in the tumor cells (*myogenin, 10.5*×). (D) Melanocytic markers, including HMB‐45, were negative (HMB‐45, 10.5×).

Gross evaluation of the excision specimen demonstrated a fungating mass measuring 8 (superior–inferior) × 7 (mediolateral) × 5.2 (superficial‐deep) cm. It extended to a depth of 1.7 cm below the skin surface, with its midpoint at 0.9 cm above the skin surface. Histopathologic examination of both specimens revealed identical findings: a sheet‐like proliferation of atypical epithelioid‐to‐rhabdoid cells with enlarged vesicular nuclei, variably prominent nucleoli, and amphophilic cytoplasm (Figure 2). A conventional melanoma component, in situ or otherwise, was not identified. The mitotic index was 32 per 2.37 mm^2^, and coagulative necrosis was present. Immunohistochemical staining was performed (Figure 2); the neoplastic cells were diffusely positive for desmin, myogenin, myoD1, and negative for cytokeratins, HMB45, S100, SOX10, BRAF V600E, and NRAS Q61R. A PRAME stain showed expression positive in > 75% of the tumor cells. Metastatic tumor was present in one axillary lymph node detected on PET‐CT and exhibited the same immunoprofile as the primary tumor.

Whole exome sequencing was performed (*Caris Life Sciences*, Phoenix, AZ). Findings included a high tumor mutational burden (TMB) of 19 mutations/Mb and a preponderance of C>T (with complementary G>A and CC>TT base pair changes). A summary of all genetic variants can be found in Table 1. After discussion at the tumor board, a diagnosis of trans‐differentiated melanoma was considered most likely, and the patient was initiated on adjuvant PD‐1 inhibitor therapy. The patient is alive with no evidence of disease at 10 months of follow‐up.

**TABLE 1:** Mutational profile of transdifferentiated melanoma.

Gene	Protein alteration	DNA alteration	Variant frequency (%)	Variant interpretation
*ARID2*	p.Q476*	c.1426C>T	37	Pathogenic variant
*BRCA2*	p.L105*	c.314T>A	10	Pathogenic variant
*CRKL*	—	—	—	Amplified
*FANCB*	c.1327‐1G>A	c.1327‐1G>A	53	Pathogenic variant
*LZTR1*	p.Q420*	c.1258C>T	88	Pathogenic variant
*PTPN11*	p.Q510L	c.1529A>T	41	Likely pathogenic variant
*TERT*	—	c.‐124C>T	24	Pathogenic variant
*APC*	p.S179F	c.536C>T	14	VUS
*EGFR*	p.E245K	c.733G>A	7	VUS
*ERBB2 (Her2/Neu)*	p.T216M	c.647C>T	8	VUS
*FLCN*	p.P63S	c.186_187 delinsTT	37	VUS
*TP53*	p.G199_N200 delinsVY	c.596_598 delinsTAT	35	VUS

*Note*: Whole exome sequencing revealed a preponderance of C>T (with complementary G>A), and CC>TT, base pair changes, indicating an ultraviolet‐induced mutational signature. Most of the variants of pathogenic significance have been reported frequently in melanoma.

Abbreviation: VUS, variant of uncertain significance.

## 3 Discussion

Trans/de‐differentiated melanomas are rare and poorly understood [2]. Classically, they are biphasic tumors composed of an element of conventional melanoma with retained melanocytic markers (e.g., SOX10, S‐100, Melan‐A, and HMB45) and an adjacent population of cytologically distinct cells that lack any evidence of melanocytic differentiation. This second population may either lack any indication of lineage differentiation (de‐differentiated) or may adopt heterologous differentiation (trans‐differentiated) [1]. In some cases, as in our case, a conventional melanoma component may be absent.

Clinically, trans‐differentiated melanoma presents with large and frequently ulcerated nodules that arise most commonly on the sun‐damaged skin of elderly men [1, 2]. Although trans‐differentiated melanoma tends to exhibit poor prognostic indicators of melanoma, its clinical behavior appears similar to that of an equivalent conventional melanoma [1].

The diagnosis of trans‐differentiated melanoma can be challenging, as an adjacent conventional melanoma component may be either difficult to identify (due to focality or extensive ulceration) or entirely absent [2]. In such cases, the presence of histopathologic features commonly associated with melanoma (such as: aggregates of tumor infiltrating lymphocytes; junctional nests of sarcomatous cells; melanin pigment; or microsatellitosis) may be helpful. Thorough tissue sampling is therefore paramount. Immunoreactivity for PRAME may be helpful in some cases [3], however, PRAME positivity was seen in a subset of rhabdomyosarcomas (69% of cases positive, with 38% exhibiting 4+ staining), representing a potential pitfall [4].

In very rare cases where none of these features are present, a high index of suspicion and subsequent molecular analysis may be necessary to make the diagnosis. Primary cutaneous rhabdomyosarcoma is exceedingly rare, with the largest series comprising only 11 cases; this was a major factor driving the decision to pursue molecular testing in the present case [5]. Interpretation of molecular profiling may be complicated by the fact that such data have only been reported in four cases of trans‐differentiated melanoma with rhabdomyosarcomatous differentiation [2, 6, 7]. While high TMB as seen in our case (19 mutations/Mb) is characteristic of cutaneous melanoma and cutaneous squamous cell carcinoma, TMB status has not been robustly studied in primary cutaneous rhabdomyosarcoma; TMB in trans‐differentiated melanomas with rhabdomyosarcomatous differentiation has been reported from 92 to 197 mutations/Mb [1, 2, 7]. A UV‐induced mutational profile, characterized by a preponderance of C>T base substitutions at dipyrimidine sites, was also demonstrated in the present case and is consistent with prior reports [2, 7]. Even so, such changes are characteristic of all tumors arising in sun‐damaged skin, including squamous cell carcinoma, melanoma, and cutaneous sarcomas [8]. Regarding the presently detected variants of pathogenic significance, melanoma was among the top 6 most common cancers to exhibit mutations in each of those genes (except *BRCA2*), which were not reported with significant frequency in rhabdomyosarcoma [9]. There was a *TERT* promoter mutation, which is a well‐characterized driver mutation in melanoma. Conversely, *TERT* promoter mutations are rare in soft tissue sarcomas and have not been reported in any unequivocal rhabdomyosarcomas [10]. Genomic events associated with rhabdomyosarcoma in other contexts, such as *MYOD1* mutations and *NCOA2* rearrangements [11], were not detected. A variety of mutations have been previously reported in trans‐differentiated melanomas with rhabdomyosarcomatous differentiation, the most common being *NF1* (4/4), *TP53* (4/4), *TERT* (2/4), *FGFR2/3* (2/4), and *CDKN2A* (2/4).

After careful consideration of the available data, our decision in the present case was to render a final diagnosis of a primary cutaneous neoplasm with rhabdomyosarcomatous differentiation. While we favor a diagnosis of transdifferentiated melanoma based on the mutational landscape, the true cell of origin of this tumor remains unclear. Consequently, the differential diagnosis also includes cutaneous squamous cell carcinoma with heterologous rhabdomyosarcomatous differentiation and primary cutaneous rhabdomyosarcoma. As rhabdomyosarcoma is one of the few sarcomas known to exhibit lymph node metastasis (which has been demonstrated also in primary cutaneous rhabdomyosarcomas) [5], the clinical behavior of this tumor did not tip the balance one way or another. It may indeed be the case that many, if not all, cases of primary cutaneous rhabdomyosarcoma are actually cases of transdifferentiated melanoma. Further studies, such as DNA methylation profiling, may further elucidate this theory [7].

Regardless, the presence of a high TMB opens the door to treatment options with immune checkpoint inhibitor therapy, which has been successful in this patient to date. Among the many FDA‐approved indications for PD‐1 inhibitors are: melanoma (adjuvant and neoadjuvant), cutaneous SCC (neoadjuvant), head and neck SCC (adjuvant), and solid tumors with high TMB (> 10 mutations/Mb) [12]. Literature on the use of PD‐1 inhibitors in rhabdomyosarcoma is limited to one trial of combination therapy with PD‐1 and CTLA‐4 inhibitors, which found a clinically significant sustained partial response in 1 of 7 pediatric patients with alveolar rhabdomyosarcoma [13].

## Ethics Statement

The authors have nothing to report.

## Conflicts of Interest

The authors declare no conflicts of interest.

## Data Availability

The authors have nothing to report.
